# Measuring the capacity of yeast for surface display of cell wall‐anchored protein isoforms by using β‐lactamase as a reporter enzyme

**DOI:** 10.1002/2211-5463.13886

**Published:** 2024-08-28

**Authors:** Tea Martinić Cezar, Antonia Paić, Stefani Prekpalaj, Renata Teparić, Bojan Žunar, Igor Stuparević

**Affiliations:** ^1^ Laboratory for Biochemistry, Department of Chemistry and Biochemistry, Faculty of Food Technology and Biotechnology University of Zagreb Croatia

**Keywords:** biocatalyst, cell wall proteins, yeast, yeast surface display, β‐lactamase

## Abstract

Yeast surface display is a promising biotechnological tool that uses genetically modified yeast cell wall proteins as anchors for enzymes of interest, thereby transforming yeast cell wall into a living catalytic material. Here, we present a comprehensive protocol for quantifying surface‐displayed β‐lactamase on the cell wall of model yeast *Saccharomyces cerevisiae*. We use β‐lactamase as a reporter enzyme, which we tagged to be anchored to the cell wall closer to its N or C terminus, through the portion of the Pir2 or Ccw12 cell wall proteins, respectively. The catalytic activity of surface‐displayed β‐lactamase is assessed by its ability to hydrolyze nitrocefin, which produces a colorimetric change that is quantitatively measured by spectrophotometric analysis at 482 nm. This system enables precise quantification of the potential of *S. cerevisiae* strains for surface display, continuous real‐time monitoring of enzyme activity, and facilitates the study of enzyme kinetics and interactions with inhibitors within the cell's native environment. In addition, the system provides a platform for high‐throughput screening of potential β‐lactamase inhibitors and can be adapted for the visualization of other enzymes, making it a versatile tool for drug discovery and bioprocess development.

## Graphical abstract

Yeast surface display (YSD) uses genetically modified yeast to immobilize recombinant enzymes on the cell surface. To quantify the efficiency of YSD, β‐lactamase was fused to selected cell wall proteins and enzyme activity was measured spectrophotometrically at 482 nm using nitrocefin as substrate. This system enables the monitoring of enzyme activity, high‐throughput screening of inhibitors, and versatile applications in bioprocess.

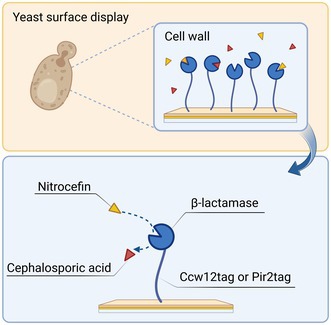

**Abbreviations**
AmpR: ampicillin resistance gene
BLA: gene coding for β‐lactamase in *Escherichia coli*
DMSO: anhydrous dimethyl sulfoxide
OD_600_
: optical density of a sample measured at a wavelength of 600 nm in 1 cm light path
YSD: yeast surface display

Yeast cells are surrounded by a protective cell wall, which is crucial for their interaction with the environment. Although the cell wall primarily consists of polysaccharides, it is also enriched with mannoproteins, which are linked to it by covalent and non‐covalent interactions [1, 2]. The cell wall's flexibility and susceptibility to numerous modifications make it suitable for biotechnological applications, including the promising yeast surface display (YSD) technique [3, 4, 5, 6].

Yeast surface display employs genetic engineering to transform native cell wall proteins into anchors for biotechnologically interesting enzymes, effectively transforming the yeast cell wall into an active living material [7, 8]. This modification ensures consistent, cost‐effective, and sustainable production of enzymes of interest, which the cell immobilizes to its outer surface, making them easily accessible to challenging substrates that cannot penetrate the cell membrane. In addition, YSD allows precise control over gene expression and protein immobilization, streamlines the product purification process, and facilitates biocatalyst reuse and recovery [9, 10]. Moreover, YSD offers several advantages over traditional enzyme immobilization techniques, which typically involve attaching enzymes to inert supports such as beads, fibers, or membranes (reviewed in Ref. [11]). Thus, YSD offers a more natural, accessible, and economical approach to enzyme immobilization, making it superior to many traditional immobilization methods.

However, despite YSD's numerous advantages, achieving sufficiently high display efficiency for industrial applications remains a challenge. To improve efficiency and create a robust YSD platform, it is necessary to optimize the genetic construct for recombinant protein expression [12], select a suitable yeast strain [13], and even consider using alternative yeast hosts [14, 15]. To ensure these optimizations are effective, comprehensive characterization of surface‐displayed and total cell wall proteins is essential.

Several methods are available for characterizing cell wall proteins. These methods vary in their specificity and the type of information they provide. One such method involves visualizing all cell wall proteins by labeling the cell surface with biotin, which is then followed by streptavidin‐based immunoblotting [16]. Alternatively, specific cell wall proteins can be visualized by tagging them appropriately and then performing anti‐tag immunoblotting. With these methods, it is also possible to determine the type of chemical bond between the protein and the cell wall. For this purpose, it is necessary to isolate the cell wall and treat it with SDS, β‐glucanase, or NaOH to extract the appropriate fraction of cell wall proteins before performing the immunoblot, which reveals whether the protein is bound to the cell wall non‐covalently or is GPI‐ or Pir‐anchored. However, these techniques generally provide only semi‐quantitative results, indicating the protein's presence in the cell wall without assessing its activity.

We addressed this problem by developing a quantitative method for measuring the enzymatic activity of surface‐displayed proteins (Fig. 1). As a reporter protein, the method employs β‐lactamase, an extensively characterized 29 kDa monomeric enzyme without potential N‐glycosylation sites or serine/threonine‐rich tracts that could be extensively O‐glycosylated [17]. As such, due to its small size and resistance to glycosylation, β‐lactamase can be secreted and covalently linked to the cell wall, without losing its activity.

**Fig. 1:**
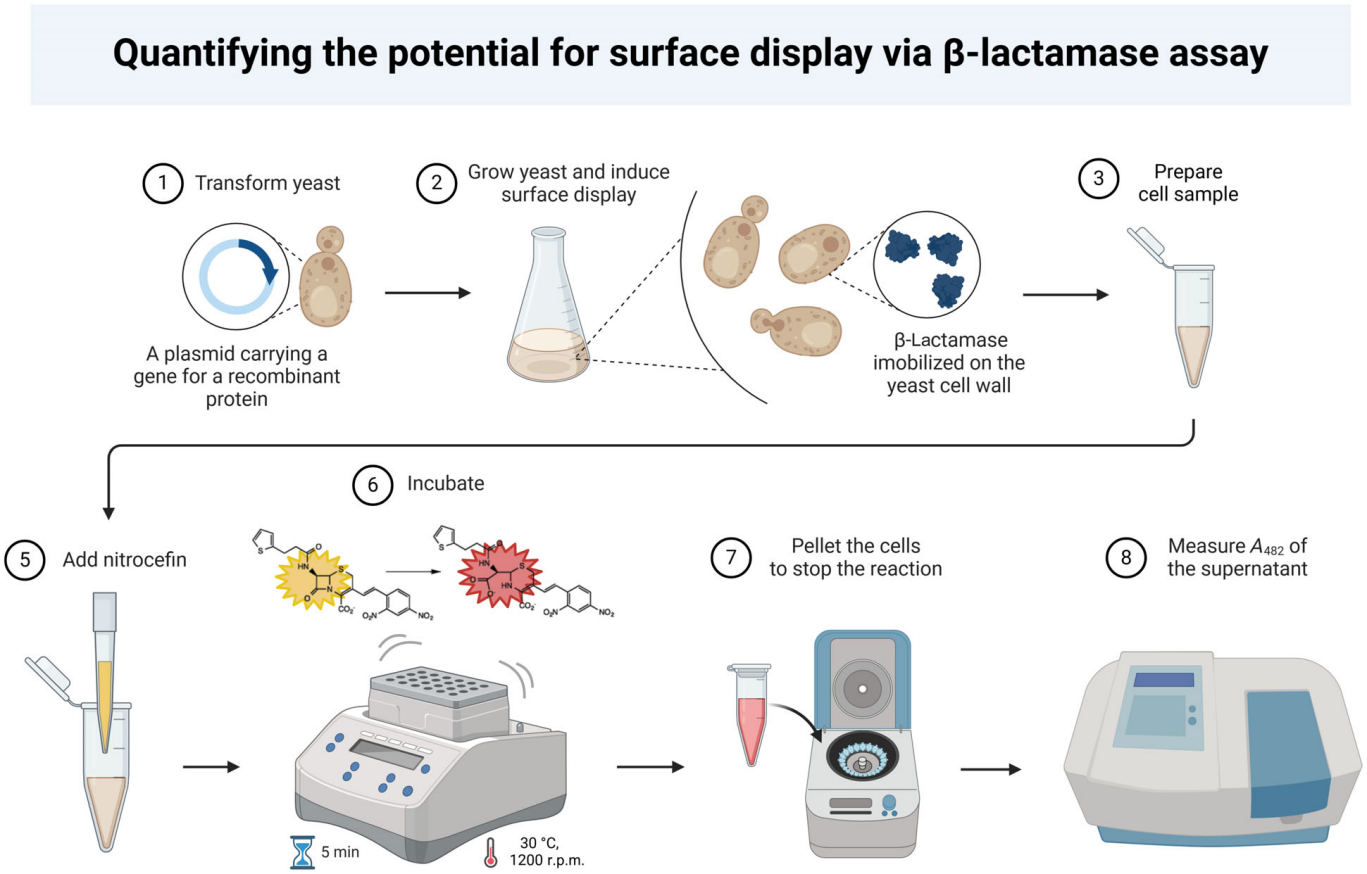
The scheme for measuring the activity of the surface‐displayed β‐lactamase.

The assay presented here enables researchers to confidently quantify the activity of β‐lactamase linked to the cell wall closer to its N or C terminus (Fig. 2). This distinction is important as some enzymes remain active only when immobilized near a specific terminus due to conformational restrictions [18, 19]. To quantify β‐lactamase bound near its N terminus, the method employs a 40 kDa Hsp150/Pir2‐β‐lactamase fusion protein Pir2tag‐bla. This fusion protein directs the reporter into the secretory system via the Pir2 signal sequence and covalently links it to the β‐1,3‐glucan through a glutamine residue within its Pir repeat. Conversely, to quantify β‐lactamase bound near its C terminus, the method uses a 40 kDa Ccw12‐β‐lactamase fusion protein Ccw12tag‐bla. This protein directs the reporter into the secretory system via the Ccw12 signal sequence and covalently links it to the β‐1,6‐glucan via the C terminus of the Ccw12 protein, which the cell transfers to the GPI anchor.

**Fig. 2:**
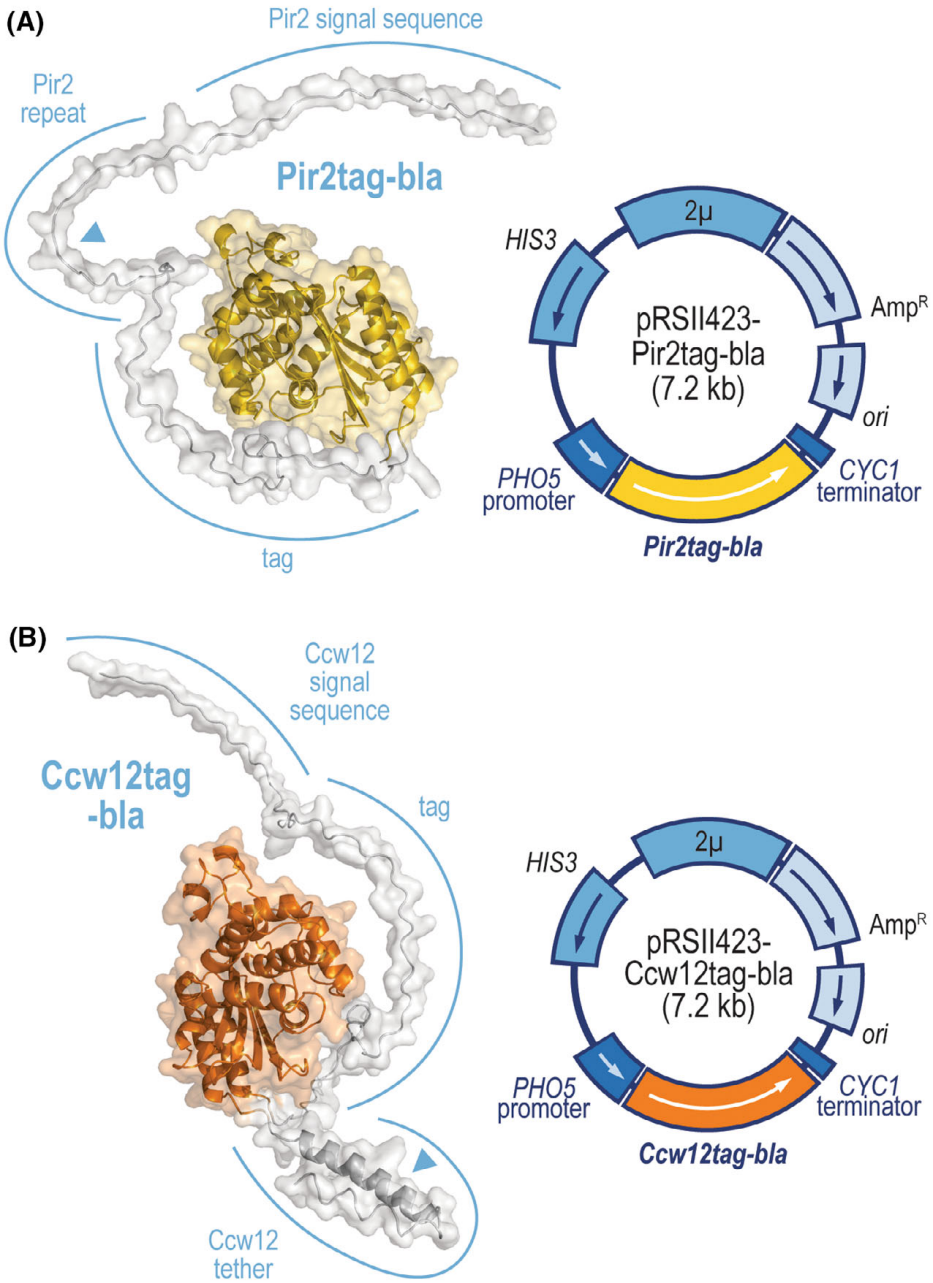
Annotated structures of proteins Pir2tag‐bla (A) and Ccw12tag‐bla (B), as well as maps of plasmids encoding them. Unstructured protein regions are shaded silver. β‐Lactamase domain is shaded yellow or orange. The triangles denote amino acid residues through which the Pir2p and Ccw12p tags covalently anchor β‐lactamase to the cell wall. The plasmids, being derived from yeast shuttle vector pRSII423, encode *Escherichia coli* plasmid replication origin (*ori*), yeast 2μ replication origin (*2μ*), *E. coli* selection marker that imparts the bacteria with resistance to ampicillin (Amp^R^), and *Saccharomyces cerevisiae HIS3* selection marker that allows yeasts to grow in media without histidine. The surface display reporter genes, encoding cell wall‐bound β‐lactamase (*Pir2tag‐bla*, *Ccw12tag‐bla*), are under the control of the *S. cerevisiae PHO5* promoter, which is a strong promoter induced in phosphate‐free media, and the *CYC1* terminator. Plasmids pRSII423‐Pir2 and pRSII423‐Ccw12 do not encode cell wall‐bound β‐lactamase and serve as negative controls, instead encoding full Pir2p protein and Ccw12p signal and anchoring sequence, respectively.

Fusion proteins Pir2tag‐bla and Ccw12tag‐bla are encoded on 2μ *HIS3* plasmids, which allows for strong reporter expression under inducible conditions. Both fusion proteins are controlled by the *PHO5* promoter, induced in a phosphate‐free medium, which avoids potential growth defects during the exponential phase that would originate from an overwhelmed secretory system. The open reading frames of the fusion proteins consist of native *Saccharomyces cerevisiae* Pir2 and Ccw12 signal and anchoring sequences fused with a *de novo* synthesized sequence encoding β‐lactamase. This β‐lactamase‐encoding sequence is codon‐usage optimized for *S. cerevisiae* and lacks CTG codons, which makes it functional even in yeasts belonging to the CTG clade.

In the described system, the catalytic activity of the surface‐displayed β‐lactamase is reflected in its ability to cleave nitrocefin, a cephalosporin derivative characterized by its yellow coloration and limited cellular uptake [20]. Enzymatic cleavage of the β‐lactam ring within the nitrocefin molecule produces cephalosporic acid, which causes a distinct red coloration and a subsequent shift in the absorption spectrum [21]. Quantification of β‐lactamase activity can be performed by spectrophotometric analysis at 482 nm and provides a reliable method for evaluating enzymatic kinetics. In this way, it is possible to monitor continuously and in real‐time the activity of the surface‐displayed enzyme. Beyond its role as a reporter enzyme, surface‐displayed β‐lactamase can be utilized to characterize and screen libraries of new β‐lactamase inhibitors [22] and to study structural and functional properties of different β‐lactamase variants. Moreover, β‐lactamase can serve as a reporter tag, enabling quantification of other surface‐displayed proteins once it is fused with them.

## Materials

- *Saccharomyces cerevisiae* strain whose potential for surface display the researcher wishes to measure.
- Plasmid pRSII423‐Pir2tag‐bla, encoding β‐lactamase that is anchored to the cell wall through the N‐terminally positioned Pir repeat, and plasmid pRSII423‐Pir2, encoding appropriate negative control (Fig. 2).
- Plasmid pRSII423‐Ccw12tag‐bla, encoding β‐lactamase, that is anchored to the cell wall through Ccw12 anchoring sequence, and plasmid pRSII423‐Ccw12, encoding appropriate negative control (Fig. 2). The Ccw12 anchoring sequence is positioned at the enzyme's C terminus.
- Phosphate‐rich chemical‐defined medium (YNBP^+^/His^−^): 20.0 g·L^−1^ glucose, 6.7 g·L^−1^ Difco yeast nitrogen base without amino acids, 1 g·L^−1^ KH_2_PO_4_, 1.6 g·L^−1^ His^−^ drop‐out. For solid media, supplement with 20.0 g·L^−1^ agar. Autoclave.
- Phosphate‐free synthetic medium (P^−^/His^−^): 20.0 g·L^−1^ glucose, 1.0 g·L^−1^ KCl, 2.0 g·L^−1^ asparagine, 0.5 g·L^−1^ MgSO_4_·7H_2_O, 0.1 g·L^−1^ NaCl, 0.1 g·L^−1^ CaCl_2_·2H_2_O, 5.9 g·L^−1^ Na‐citrate, 2 g·L^−1^ His^−^ drop‐out, 2 mL·L^−1^ trace element stock solution. Adjust pH to 5.5 with concentrated HCl. Autoclave. Sterilely add 4 mL·L^−1^ riboflavin stock solution and 1 mL·L^−1^ biotin stock solution after the medium has cooled.
- His^−^ drop‐out: 3.0 g adenine, 2.0 g l‐alanine, 2.0 g l‐arginine, 2.0 g l‐asparagine, 2.0 g l‐aspartic acid, 2.0 g l‐cysteine, 2.0 g l‐glutamine, 2.0 g l‐glutamic acid, 2.0 g l‐glycine, 2.0 g inositol, 2.0 g l‐isoleucine, 4.0 g l‐leucine, 4.0 g l‐lysine, 2.0 g l‐methionine, 0.2 g *p*‐aminobenzoic acid, 2.0 g l‐phenylalanine, 2.0 g l‐proline, 2.0 g l‐serin, 2.0 g l‐threonine, 2.0 g l‐tryptophane, 2.0 g l‐tyrosine, 2.0 g uracil, 2.0 g l‐valine.
- Trace element stock solution: 4 mm boric acid, 0.08 mm CuSO_4_·5H_2_O, 0.3 mm KI, 0.4 mm FeCl_3_·6H_2_O, 0.7 mm MgSO_4_·7H_2_O, 0.08 mm (NH_4_)_6_Mo_7_O_24_·4H_2_O, 0.7 mm ZnSO_4_·7H_2_O. Autoclave.
- Riboflavin stock solution: 0.13 mm riboflavin, 0.3 mm *p*‐aminobenzoic acid, 2.8 mm inositol. Sterilize by filtration.
- Biotin stock solution: 0.08 mm biotin, 8.0 mm Ca‐pantothenate, 32.5 mm nicotinic acid, 20 mm pyridoxal‐HCl, 12 mm thiamine‐HCl, 0.05 mm folic acid. Sterilize by filtration.
- 50 mm K‐phosphate buffer, pH 7: 9.34 g·L^−1^ K_2_HPO_4_, 6.31 g·L^−1^ KH_2_PO_4_. Adjust pH to 7. Autoclave.
- 1 mm Nitrocefin solution: dissolve 5 mg nitrocefin (Sigma‐Aldrich, St. Louis, MO, USA, SAD) in 0.5 mL DMSO and dilute with 50 mm K‐phosphate buffer (pH 7) to 1 mm nitrocefin. Store at −20 °C.
- Themoshaker, centrifuge, spectrophotometer.

## Methods

Transform the strain of *S. cerevisiae* whose potential for surface display strain one wishes to determine with plasmids pRSII423‐Pir2tag‐bla, pRSII423‐Pir2, pRSII423‐Ccw12tag‐bla, and pRSII423‐Ccw12. The strain should lack a functional *HIS3* gene. For this procedure, we regularly use the protocol described by Gietz and Schiestl [23], although any standard yeast transformation protocol should suffice.

### Inducing the *PHO5* promoter

1. Inoculate yeast cells into 5 mL of YNBP^+^/His^−^ medium in a sterile 50 mL Falcon tube and grow overnight at 30 °C/180 r.p.m. Grow two cultures for each strain, i.e., perform the assay in at least biological duplicates.
2. The next morning, the cells should be in the stationary phase (OD_600_ per mL = 5–6). Measure the OD_600_ and transfer 7.5 OD_600_ of cells into a new sterile 50 mL Falcon tube. Add fresh YNBP^+^/His^−^ medium to the final volume of 15 mL, to obtain 0.5 OD_600_ per mL cell density.
3. Grow at 30 °C/180 r.p.m. until the culture reaches the mid‐exponential phase of 2 OD_600_ per mL (approximately 4.5 h).
4. Transfer 4.5 OD_600_ of cells to a new sterile 50 mL Falcon tube. Centrifuge at 850 ***g***/5 min, discard the supernatant and wash the cells with 15 mL sterile deionized water (sdH_2_O) to remove excess phosphate. To induce the *PHO5* promoter, resuspend the cells in 15 mL P^−^/His^−^ medium to a final concentration of 0.3 OD_600_ per mL. Incubate overnight at 30 °C/180 r.p.m. By the next morning, the cells should reach the stationary phase (approximately 2 OD_600_ per mL).

### Concentrating the cells

1. In the morning, measure the OD_600_ per mL of the overnight P^−^/His^−^ cultures. The value should not exceed 2 OD_600_ per mL.
2. Centrifuge the yeast cultures at 850 ***g***/5 min and discard the supernatant.
3. Wash the cell pellet in 15 mL sdH_2_O, centrifuge at 850 ***g***/5 min, and discard the supernatant.
4. Wash the cell pellet in 15 mL 50 mm K‐phosphate buffer (pH 7), centrifuge at 850 ***g***/5 min, and discard the supernatant.
5. Resuspend the cell pellet in 1 mL 50 mm K‐phosphate buffer (pH 7) and transfer the suspension into a 1.5 mL Eppendorf tube.
6. Centrifuge cell suspension at 6000 ***g***/3 min and discard the supernatant.
7. Adjust the cell concentration to approximately 80 OD_600_ per mL.
8. Measure the exact OD_600_ per mL of the 160× diluted cell suspension (dilute 5 μL of suspension from step 11 with 795 μL sdH_2_O, measure OD_600_ per mL, and multiply the calculated value with 160).
9. Prepare the cell stock by diluting 10 μL of the cell suspension (approximately 80 OD_600_ per mL) with 90 μL of K‐phosphate buffer (50 mm, pH 7).

### Enzyme assay

1. Prepare the reaction mixtures without substrate and incubate 2 min/30 °C/1200 r.p.m. on a thermoshaker. For each cell stock, perform three nitrocefin reactions in parallel, i.e., measure technical triplicates for each biological replicate.
  - blank: 475 μL K‐phosphate buffer (50 mm, pH 7)
  - reaction: 467.5 μL K‐phosphate buffer (50 mm, pH 7) + 7.5 μL cell stock (from step 13)
2. Add 25 μL nitrocefin (1 mm) to each reaction mixture and incubate 5 min/30 °C/1200 r.p.m. on a thermoshaker.
3. To stop the reaction, pellet the cells by centrifuging them (30 s/6000 ***g***).
4. Transfer the 350 μL of supernatant into cuvettes and measure the *A*_482_, using the above blank to zero the spectrophotometer.

### Data analysis

1. For each technical replicate, calculate the following value of *A*_482_/OD_600_:

$$\;A_{482}/{\mathrm{OD}}_{600}=A_{482}/\left({\mathrm{OD}}_{600\;\left(\text{from step}\;12\right)}\cdot 160\cdot 7.5\times 10^{-4}\right)$$

The factor of 160 corrects for diluting 5 μL of cells in 795 μL sdH_2_O, while the factor of 7.5 × 10^−4^ corresponds to the volume (7.5 μL) of a 10‐fold diluted cell suspension added to the reaction.

1. Calculate the mean of these values for each set of technical triplicates (Pir2tag‐bla, Pir2, Ccw12tag‐bla, Ccw12).
2. Subtract from the mean of Pir2tag‐bla or Ccw12tag‐bla reactions the mean of appropriate negative controls (Pir2 or Ccw12, respectively).

## Tips & Tricks

1. To prepare and store a 1 mm nitrocefin solution, dissolve 5 mg of nitrocefin powder in 0.5 mL of DMSO and vortex thoroughly to dissolve completely. Divide the solution into 100 μL aliquots and store at −20 °C to maintain stability. Thaw a 100 μL aliquot immediately before use and dilute with 1.9 mL of 50 mm phosphate buffer (pH 7). This dilution gives a ready‐to‐use solution for about approximately 80 reactions. Such procedures for handling and storing nitrocefin avoid the formation of orange difficult‐to‐dissolve nitrocefin particles. Since nitrocefin is light‐sensitive, store it in light‐impenetrable tubes. Moreover, avoid repeated freeze–thaw cycles to prevent its degradation.
2. The described protocol lists values and dilutions that should produce *A*_482_ values of approximately 0.5, when measured with Eppendorf BioSpectrometer Basic. The resulting change in color is also clearly visible to the naked eye.
3. In the standard laboratory *S. cerevisiae* strain BY 4741, we observe that the surface display of Pir2tag‐bla is 50–80% as effective as that of Ccw12tag‐bla.
4. A potential problem may arise when evaluating the efficiency of YSD in flocculation‐prone strains, whose OD_600_ values are challenging to measure properly. To avoid this, pretreat these strains with EDTA or mannose to break the flocs and obtain single cells [24].
5. This protocol also allows measuring the activity of the reporter proteins secreted in the media. For this purpose, after cell centrifugation in step 6, transfer the supernatant to a new sterile Falcon tube. To perform the activity assay, mix 220 μL of the media, 255 μL of K‐phosphate buffer (50 mm, pH 7), and 25 μL of nitrocefin solution (1 mm). The reaction conditions are the same as for the cell‐based assays.
6. While this protocol focuses on nitrocefin, other colorimetric and fluorogenic β‐lactamase substrates that could be used do exist [17, 25, 26]. Moreover, the here‐described assay could, in principle, be converted to an agar plate assay to allow for high‐throughput strain and mutant screening [27].

## Conclusion

We present a step‐by‐step, user‐friendly protocol for quantifying yeast's potential for surface display. By using β‐lactamase‐based fusion proteins targeting the cell wall, this assay efficiently complements other cell wall and YDS characterization methods. As such, this method can be used to advance the dissection of molecular mechanisms orchestrating the secretion of native and biotechnologically interesting proteins, as well as improve YSD.

## Conflict of interest

The authors declare no conflict of interest.

## Author contributions

TMC, AP, and SP acquired the data; TMC, AP, and BŽ analyzed and interpreted the data; IS, RT, and BŽ wrote the paper; IS and RT conceived and designed the project.

## Data Availability

Plasmids pRSII423‐Pir2tag‐bla, pRSII423‐Pir2, pRSII423‐Ccw12tag‐bla, and pRSII423‐Ccw12 are available upon request.
